# A Comprehensive Characterisation of Volatile and Fatty Acid Profiles of Legume Seeds

**DOI:** 10.3390/foods8120651

**Published:** 2019-12-06

**Authors:** Prit Khrisanapant, Biniam Kebede, Sze Ying Leong, Indrawati Oey

**Affiliations:** 1 Department of Food Science, University of Otago, PO Box 56, Dunedin 9054, New Zealand; prit.khrisanapant@postgrad.otago.ac.nz (P.K.); biniam.kebede@otago.ac.nz (B.K.); sze.leong@otago.ac.nz (S.Y.L.); 2 Riddet Institute, Private Bag 11 222, Palmerston North 4442, New Zealand

**Keywords:** legumes, volatiles, fatty acids, characterisation, fingerprinting, multivariate data analysis

## Abstract

Legumes are rich in unsaturated fatty acids, which make them susceptible to (non) enzymatic oxidations leading to undesirable odour formation. This study aimed to characterise the volatile and fatty acid profiles of eleven types of legumes using headspace solid-phase microextraction gas chromatography–mass spectrometry (HS-SPME-GC-MS) and GC coupled with a flame ionisation detector (GC-FID), respectively. Volatile aldehydes, alcohols, ketones, esters, terpenes and hydrocarbons were the chemical groups identified across all the legumes. The lipids comprised palmitic, stearic, oleic, linoleic and α-linolenic acids, with unsaturated fatty acids comprising at least 66.1% to 85.3% of the total lipids for the legumes studied. Multivariate data analysis was used to compare volatile and fatty acid profiles between legumes, which allow discriminant compounds pertinent to specific legumes to be identified. Results showed that soybean, chickpea and lentil had distinct volatile and fatty acid profiles, with discriminating volatiles including lactone, ester and ketone, respectively. While all three *Phaseolus* cultivars shared similar volatile profiles, 3-methyl-1-butanol was found to be the only volatile differentiating them against the other eight legumes. Overall, this is the first time a multivariate data analysis has been used to characterise the volatile and fatty acid profiles across different legume seeds, while also identifying discriminating compounds specific for certain legume species. Such information can contribute to the creation of legume-based ingredients with specific volatile characteristics while reducing undesirable odours, or potentially inform relevant breeding programs.

## 1. Introduction

The seeds of legume plants (usually referred to as ‘legumes’) are a nutritious source of proteins, carbohydrates, lipids, vitamins and minerals [1]. Unprocessed legumes have a distinct odour due to their inherent plant metabolism [2]. Regrettably, legumes are not widely utilised to their full potential due to various factors, such as low protein digestibility, their hard-to-cook-effect and undesirable odours. The undesirable odours are largely influenced by lipoxygenase-catalysed unsaturated fatty acid oxidation that occurs during several postharvest processes [3,4]. Trained sensory panels have used negative descriptors such as beany, musty, haylike, grassy and green to describe the odours of legumes such as soybean and peas. It has been recognised that these odours are majorly derived from volatile compounds such as hexanal and 1-octen-3-ol [3,4,5]. Previous studies [3,6,7,8,9] have reported volatile compounds and their formation mainly in soybean, and there is still a limited understanding on the volatile profile of other commercially relevant legumes such as cowpea, lentil, common bean and pea [10,11,12,13]. However, these studies used targeted analysis without providing a holistic picture of all low molecular weight compounds present in the legume’s volatile fraction. Advancements in volatile analysis and instrument sensitivity are able to capture a wider range of volatile compounds; hence, they enable researchers to take an untargeted fingerprinting approach. By definition, fingerprinting is an untargeted analytical approach aiming to detect as many compounds as possible in a particular food fraction [14]. This untargeted fingerprinting approach has been used as a tool to study the effect of cooking on legumes [10,15]. To date, there is still a gap in the detailed, holistic characterisation of legume volatiles using this approach, and hence, it is essential to examine whether this analytical approach could differentiate different types of legumes. As previously mentioned, undesirable legume volatiles are primarily formed from fatty acid oxidation. In spite of this connection, no previous investigation has examined fatty acid composition of legumes in conjunction with their volatile profile. There is an opportunity here to elucidate the connection of fatty acid composition and volatile profile in the whole bean matrix.

Therefore, the objective of the present study was to comprehensively characterise the volatile and fatty acid profiles in commercially relevant legumes. The headspace solid phase-gas chromatography–mass spectrometry (HS-SPME-GC-MS) approach was implemented to detect volatile compounds, whereas the fatty acid was profiled using gas chromatography flame ionisation detection (GC-FID). The novelty of this study lies on the fact that (i) the fingerprinting approach is being applied for the first time on a wide range of commercial relevant legumes, and (ii) volatile and fatty acid components are analysed in an integrated fashion using multivariate data analysis. 

## 2. Materials and Methods

### 2.1. Raw Material Handling and Storage

Eleven types of dry legume seeds, namely, soybean (*Glycine max*), pea (*Pisum sativum*), chickpea (*Cicer arretium* var. kabuli), orange lentil (*Lens culinaris*), mung bean (*Vigna radiata*), fava bean (*Vicia faba*), cowpea (*Vigna unguiculata*), adzuki bean (*Vigna angularis*), kidney bean (*Phaseolus vulgaris*), navy bean (*Phaseolus vulgaris*) and black bean (*Phaseolus vulgaris*), were purchased in a single batch from the local market in Dunedin (New Zealand). Seeds with physical damages and discolouration were discarded. The remaining seeds were vacuum packed in opaque aluminium bags and stored at 4 °C until analysis.

### 2.2. Sample Preparation

Legume seeds (30 g) were ground using a laboratory blender (Waring, Auckland, New Zealand) for 60 s, with a pause every 30 s, at room temperature (20 ± 2 °C). The resulting flour was sieved to pass through an 850 µm mesh. Flour retaining between 450 and 850 µm mesh size was used for lipid analysis for consistent extraction of lipids. For volatile profiling, the grounding time was increased to 180 s to maximise flour surface area.

### 2.3. Moisture Determination

The legume flour sample (0.2 g) was weighed and transferred into a glass petri dish (Steriplan, Kimax, Auckland, New Zealand). The dish was subsequently covered with perforated aluminium foil. The drying was carried out at 130 °C in a convection oven for 16 h (Qualtex, Andrew Thom, Sydney, Australia). The samples were removed and cooled in a desiccator lined at the bottom with silica beads. The percentage of moisture content was estimated based on the weight loss after drying and cooling. The moisture content determination was conducted in five independent replicates.

### 2.4. Headspace Volatile Analysis with HS-SPME-GC-MS Fingerprinting

Headspace solid phase micro-extraction gas chromatography mass spectrometry (HS-SPME-GC-MS) was conducted according to the work of Liu and others [16] with modifications, consisting of sample preparation, incubation, volatile extraction, injection and GC-MS analysis. Prior to analysis, method parameters were optimised in order to capture a wide range of volatile compounds. The optimisation included sample weight, sample dilution, sample to salt ratio and type of GC column.

Upon sample analysis, legume flour was weighed (2.5 g) into a 20 mL glass vial, and 5 mL of saturated sodium chloride solution (360 g/L) was added to increase the solution’s ionic strength and drive the legume volatiles into the headspace. The vial was then tightly sealed with PTFE-coated silicon septa screw cap (Supelco, Sigma-Aldrich, St. Louis, MO, USA). The sealed vials were then vortexed for 30 s. 

Using the Gerstel MPS Maestro autosampler (Gerstel, Linthicum Heights, MD, USA), each sample was incubated at 40 °C for 5 min, with agitation at 250 rpm. Thereafter, headspace volatile compounds were extracted using headspace-solid phase microextraction (HS-SPME). A preconditioned (according to the manufacturer’s instructions) SPME fibber with a 30/50 μm divinylbenzene/carboxen/polydimethylsiloxane (DVB/CAR/PDMS) sorptive coating (Stableflex, Supelco, Bellefonte, PA, USA) was used to extract a wide range of volatile compounds from the headspace of the vial for 30 min at 40 °C.

For the GC-MS analysis (Agilent 6890N, Agilent Technologies, Santa Clara, CA, USA), extracted volatiles were desorbed in the injection port at 230 °C for 2 min, then injected in splitless mode onto a ZB-Wax capillary column (30 m × 0.25 mm × 0.25 μm; Agilent Technologies, Santa Clara, CA, USA) for separation with helium as the carrier gas at 1.5 mL/min. To facilitate separation and elution of the injected headspace volatile compounds, the GC oven was maintained at 50 °C for 5 min before the temperature was ramped up to 210 °C at 5 °C/min, after which it was again ramped to 240 °C at the rate of 10 °C/min, for a total GC-MS run time of 37 min. For the MS, the quadrupole was set at 70 eV, and the ion sources were 150 °C and 230 °C, respectively, with a mass-to-charge ratio scanning range of 30–300 m/z. Thereafter, the SPME fibre was regenerated according to the manufacturer’s instruction. The same SPME fibre was used across all samples. The volatile profiling for each legume seed was conducted in five independent replicates.

With regard to preprocessing of GC-MS chromatograms, volatile fingerprinting chromatograms often contain co-eluting peaks, which can confound data analysis. Therefore, an automated mass deconvolution and identification system (AMDIS; version 2.72, build 140.24, Agilent Technologies, Santa Clara, CA, USA) was used to deconvolute potential co-eluting peaks. The spectra obtained were further processed by mass profiler professional (MPP; version 14.9.1, build 1316, Agilent Technologies, Santa Clara, CA, USA), a peak filtering and alignment software. This creates aligned peaks lacking nonreproducible and background peaks. Afterwards, a table of retention time and volatile amount expressed as peak area was obtained.

Tentative identification of volatile compounds was conducted manually. In the present work, three criteria were employed to increase the power of compound identification: (i) match and reverse match with the NIST library of no less than 90%; (ii) comparison of experimental retention index with RI according to literature; and (iii) matching retention time and spectra with authentic standards from chemical groups of detected volatiles (alcohol, aldehyde, terpene and acid) (See Table S1).

### 2.5. Determination of Fatty Acids in Legume Seeds Using FAME-GC-FID

For fatty acid analysis, legume lipid was extracted and converted to fatty acid methyl esters (FAME) and detected using gas chromatography flame ionisation detection (GC-FID) according to AOAC method 963.22 [17] with modifications.

#### 2.5.1. Total Lipid Extraction Based on Soxhlet Method

Legume flour sample (2 g) was weighed and placed inside a cellulose extraction thimble (26 × 60 mm, Whatman, Buckinghamshire, UK). The filled extraction thimble was fitted onto a Soxtec distillation apparatus (Tecator, Hilleroed, Denmark). Meanwhile, aluminium cups were filled with five to ten antibumping granules and weighed before adding with 25 mL of organic solvent mix consisting of a 2:1 (*v*/*v*) ratio of chloroform (EMPARTA, Merck, Darmstadt, Germany) and methanol (Ajax Univar, North Shore, New Zealand). The cups were then fitted underneath the thimble and above the heating plate of the Soxtec distillation apparatus. Continuous reflux distillation of the samples was then carried out for 1 h with the heating plate set at 160 °C. After that, solvent was evaporated from the sample, and then the cups were released from the apparatus, and the residual solvent was allowed to evaporate inside a convection oven (Sanyo MOV-212F, New South Wales, Australia) set at 50 °C for 15 min.

The lipid yield of each sample was estimated by weight difference between the weight of the empty cups (filled with antibumping granules) and the weight of the cup filled with lipids upon completion of the solvent extraction. After weighing, 15 mL of hexane (Ajax Finechem, Auckland, New Zealand) was used to resuspend the lipids in each aluminium cup. The hexane containing lipid solution was then stored in a refrigerator at 4 °C in a tightly sealed glass tube before proceeding to the lipid purification step. The lipid extraction of each legume seed was conducted in four independent replicates.

#### 2.5.2. Lipid Purification

Fatty acid methyl esters (FAMEs) were obtained by purifying lipids by saponification to remove nonsaponifiable materials, followed by esterification into FAMEs. A volume of lipid containing hexane solution equivalent to 5 mg lipid was pipetted into a sealable glass tube. Thereafter, 5 mL of a solution containing potassium hydroxide (0.5 M, AnalaR, Leuven, Belgium) dissolved in methanol was added, and the tube was sealed immediately. The fatty acid saponification was carried out for 20 min at 80 °C on a heating block. The tubes were removed from the heating block and allowed to cool in ambient air for 10 min.

To the cooled solution, 3 mL of diethyl ether (LabServ, Auckland, New Zealand) and 7 mL of milliQ water were added, and the test tube was inverted to mix. The mixture was allowed to stand for 2 min to allow separation between water and organic solvent layers. The top layer of diethyl ether was then discarded to remove any nonsaponifiable material. The fatty acids were liberated by neutralisation to ~pH 7 with concentrated hydrochloric acid (37%; EMSURE, Merck, Darmstadt, Germany). Then, another 4 mL of diethyl ether was added and inverted to mix. The top diethyl ether layer formed was collected in a clean glass test tube for derivatisation.

#### 2.5.3. Lipid Derivatisation to Fatty Acid Methyl Esters (FAMEs)

One millilitre of boron trifluoride (14%) in methanol (Sigma-Aldrich, St. Louis, MO, USA) was promptly added as derivatisation agent. Fatty acid esterification was carried out for 20 min at 80 °C on a heating block and then cooled. After cooling, 7 mL of saturated sodium chloride solution (360 g/L) was added and vortexed for 15 s. The top diethyl ether formed was then collected for FAME GC-FID analysis.

#### 2.5.4. Fatty Acid Profiling Using GC-FID

For fatty acid analysis, gas chromatography flame ionisation detection (GC-FID) was conducted [17] with modifications. A GC-FID system (6890A G1530A; Agilent Technologies, Santa Clara, CA, USA) was used. It was equipped with an autosampler (7683 series injector, Agilent Technologies, Santa Clara, CA, USA) and fitted with a BPX70 capillary column (70% Cyanopropyl Polysilphenylene-siloxane, SGE, Victoria, Australia).

Samples (1 µL) were injected in split mode (20:1 ratio) at 240 °C for separation with hydrogen gas at 2.2 mL/min. To ensure good separation of fatty acid methyl esters, the GC oven temperature was increased from its initial temperature of 120 °C to 225 °C at the rate of 3 °C/min, then ramped to 245 °C at 10 °C/min. Once the GC oven temperature reached 245 °C, the column was held at this temperature for another 2 min. For the FID, the detector temperature was set at 250 °C.

#### 2.5.5. Identification and Data Preprocessing of FAME

Chromatograms obtained from GC-FID were analysed with GC ChemStation (Build 4.01, Agilent Technologies, Santa Clara, CA, USA) and individual peaks manually identified by matching retention time with commercial standards (FAMQ-005, AccuStandards, New Haven, CT, USA). Following manual peak alignment and removal of interfering background compounds, the proportion of signal abundance of each fatty acid was calculated in % abundance of total signal abundance. Thereafter, a table of fatty acid profile for each legume was obtained.

### 2.6. Multivariate Data Analysis and Identification of Compounds Relevant to Specific Legume Type

Multivariate data analysis, marker selection and marker identification were performed on the combined data sets comprising legume fatty acid and headspace volatile data sets.

Using both volatile and fatty acid data, multivariate data analysis was conducted using principle component analysis (PCA), followed by partial least square discriminant analysis (PLS-DA), utilising Solo software (Version 8.2.1, Eigenvector Research, Manson, WA, USA). Firstly, PCA was used as an unsupervised, exploratory technique to determine grouping/separation in the data, as well as to detect outlier. Secondly, PLS-DA was used as a supervised technique to detect similarities and differences between different legume seeds, as well as correlation between volatile compounds and legumes. Thereafter, a bi-plot was generated as a visual representation of the information obtained (OriginPro, OriginLab, Northampton, MA, USA).

Volatile compounds that showed a clear discriminant correlation with each legume were selected through determination of variable identification (VID) coefficients [18]. VID values are the corresponding correlation coefficients between X-variables (volatile compounds and fatty acids) and predicted Y-variables (Legume type). An absolute threshold value of |0.800| was selected. Therefore, volatiles with an absolute VID coefficient higher than 0.800 were plotted as bar graphs, and statistical significance between the means was determined using analysis of variance, conducted through SPSS Statistics (IBM, Version 26), followed by Tukey’s post-hoc test (*p* < 0.05). Those compounds were considered important discriminant compounds associated with each legume seed.

Discriminant volatile compounds were identified by comparing the deconvoluted mass spectra with an established mass spectra library using National Institute of Standards and Technology (NIST) Mass Spectral Search Program (Version 2.2, build June 10, 2014). The identities were also rechecked with a minimal 90% match and reverse match on NIST, as well as comparison of retention index with literature.

## 3. Results

### 3.1. Moisture Content of Legume Seeds

The moisture content of legume seeds was within the range of 7.4% (soybean) to 14.9% (kidney bean). Pea, navy bean, orange lentil and chickpea contained 9.9%, 9.6%, 8.7% and 8.4% moisture, respectively. In comparison, adzuki bean, black bean, fava bean, mung bean and cowpea were slightly moister, at 12.4%, 11.3%, 11.0%, 10.3% and 10.3%, respectively. 

### 3.2. Fatty Acid Analysis of Legume Seeds

GC-FID analysis of the fatty acid methyl esters was able to detect five clearly separated peaks in eleven legume samples. The legume lipid fractions consisted of palmitic (C16:0), stearic (C18:0), oleic (C18:1), linoleic (C18:2) and α-linolenic (C18:3) acids (Table 1). A commonality of the lipid profiles is that the level of saturated fatty acids such as palmitic and stearic acids is low, with the ratio of saturated to unsaturated fatty acids at 1:2 in cowpea and mung bean, and up to 1:5.4 and 1:6 in soybean and chickpea. On the other hand, all legumes had a high level of essential polyunsaturated fatty acids, namely, linoleic and α-linolenic, ranging from 47.3% in orange lentil to 71.0% in black bean. Out of the eleven types of legumes, four of them, i.e., chickpea, orange lentil, pea and fava bean, had a high (>20%) proportion of oleic acid, a monounsaturated fatty acid. This affirms that legumes are a good source of unsaturated fatty acids [19], with their fatty acid profile favourable from a cardioprotective perspective [20]. 

### 3.3. Volatile Analysis of Legume Seeds

**Figure 1:**
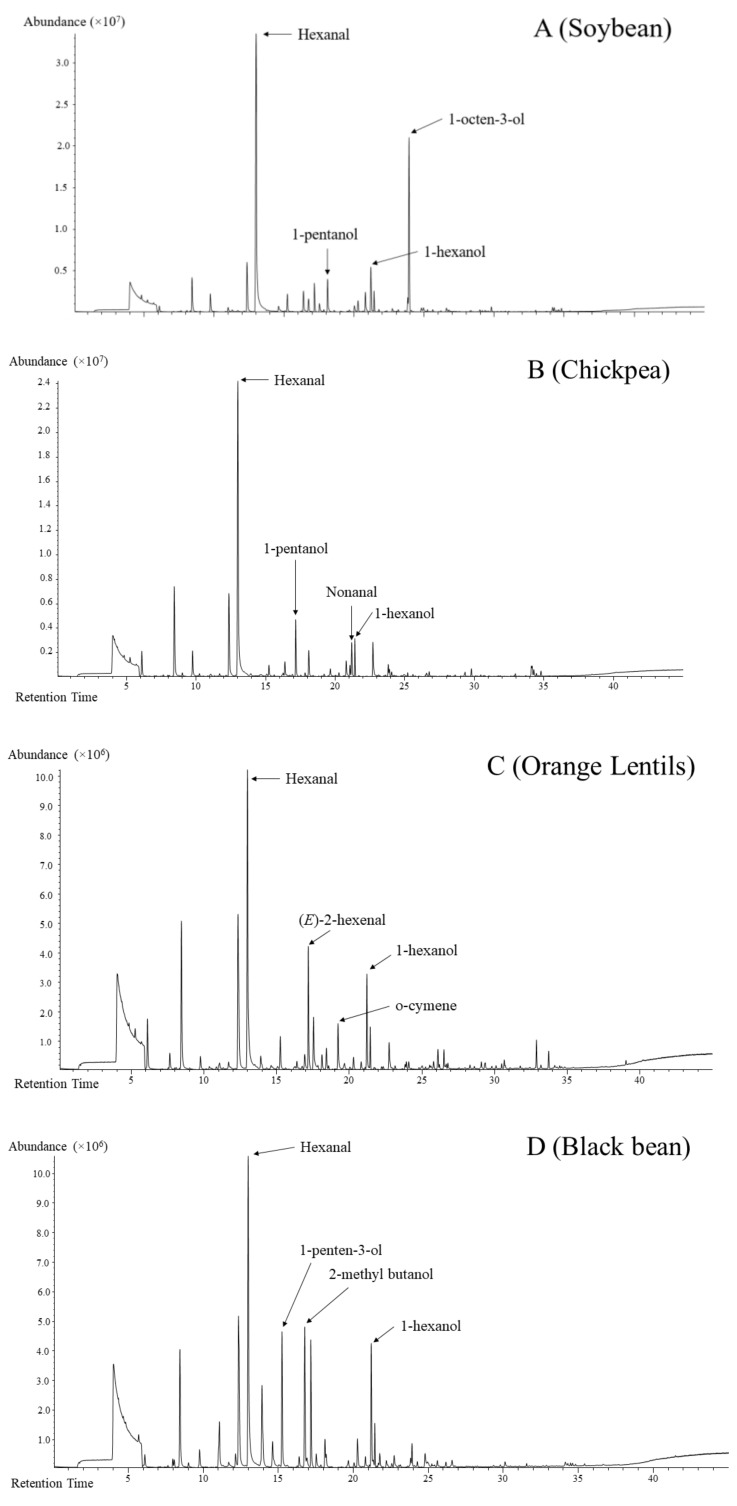
Representative total ion chromatograms of soybean (**A**), chickpea (**B**), orange lentil (**C**) and black bean (**D**) obtained with the headspace solid-phase microextraction gas chromatography–mass spectrometry (HS-SPME-GC-MS) fingerprinting method.

The HS-SPME-GC-MS fingerprinting method was able to detect an increased number of volatile compounds, totalling 97 different volatiles across all 11 legumes. Visually, the total ion chromatograms appear to be different based on the number and intensity of the peaks present amongst the samples. Some representative total ion chromatograms of the samples are shown in Figure 1.

The chemical classes of detected volatile compounds consisted of alcohols, aldehydes, ketones, esters, lactones, terpenes, hydrocarbons, furans, pyrroles and sulphur-containing compounds. Note that the percentage of specific compounds mentioned in this section refers to their relative abundance, not absolute concentration. It is also important to note that soybean and chickpea had volatiles with the highest total peak area compared to other legumes.

In soybean, a total of 63 volatile compounds were detected with the headspace fingerprinting method, consisting mainly of aldehydes, ketones, alcohols, esters, furans and sulphur and hydrocarbons. Hexanal (40.9%), 1-octen-3-ol (21.1%) and 1-hexanol (5.9%) were volatiles with the highest abundance. A total of 76 compounds were detected in chickpea with aldehydes and alcohols as two dominant chemical classes, consisting of hexanal (56.3%), nonanal (8.7%) and 1-hexanol (4.4%). Acid, ketones, furans, esters and terpenes were also present, with hexanoic acid (1.7%) being the most abundant. In cowpea, 80 headspace volatiles were detected in this study. Hexanal (22.6%), 4,1 methylethyl benzaldehyde (22.0%) and 1-hexanol (10.4%) were prominent. Other aldehydes, terpene and benzene compounds were also detected in cowpea. A total of 65 compounds were detected in pea. Alcohols and aldehydes are the majority of volatile detected in pea, such as hexanal (42.5%), 1-penten-3-ol (9.0%), 1-hexanol (7.7%) and nonanal (6.0%). In orange lentil, 82 volatile compounds were detected. Hexanal (32.7%), 1-hexanol (16.4%), 2-hexenal (5.6%) and o-cymene (4.6%) make up the top four most abundant volatile compounds, accounting for 59.3% of total volatile detected. Uniquely, lentil had the largest number (13) of terpenes of all the samples. Headspace volatiles detected in mung bean (63) comprised aldehydes, alcohols, terpenes, ketones and sulphur compounds. The top three volatiles consisted of hexanal (38.8%), 1-hexanol (13.9%) and 1, 3-dimethyl-benzene (6.3%). Interestingly, mung bean had the highest abundance of xylene (5.7%) detected out of all samples. Fava bean had the fewest (55) volatiles detected in the headspace. Aldehydes and alcohols are the main volatile detected, with terpenes, terpene derivatives, furans and esters comprising a minor fraction. Hexanal (40.4%), 3-methylbutanol (19.1%) and 3-methylbutanoic acid (10.3%) were the top three volatile compounds detected in the fava bean headspace fraction. Major volatiles detected in adzuki bean (67) headspace fraction consisted of aldehyde, alcohol and, interestingly, furans, including hexanal (21.1%), 3-furaldehyde (19.5%), 3-furanmethanol (7.5%) and 1-hexanol (6.0%). Black, navy and kidney beans had 68, 76 and 72 volatile compounds detected in the headspace, respectively, with the most abundant fraction being aldehyde and alcohol, followed by terpenes, acids and ketones. Hexanal, 1-hexanol, 1-penten-3-ol and 3-methylbutanol are similarly the major volatile compounds detected in the three *Phaseolus* samples. 

### 3.4. Comparison of the Volatile and Fatty Compositions Among the Eleven Legumes and Identifying Discriminant Compounds

Multivariate data analysis (MVDA), which is an advanced chemometrics technique, was used to compare the volatile and fatty acid profiles among the 11 legume samples and identify discriminating compounds. In order to investigate the interdependence and relation among the measured attributes, the volatile and fatty acid data were merged into a single data matrix and analysed with MVDA. A principle component analysis (PCA) was first used as an unsupervised exploratory technique to detect groupings, separations or outliers within the volatile and sample data. From the PCA modelling (results not shown), it was able to be determined that there is indeed some distinct grouping and separation within the samples and that there were no outliers.

**Figure 2:**
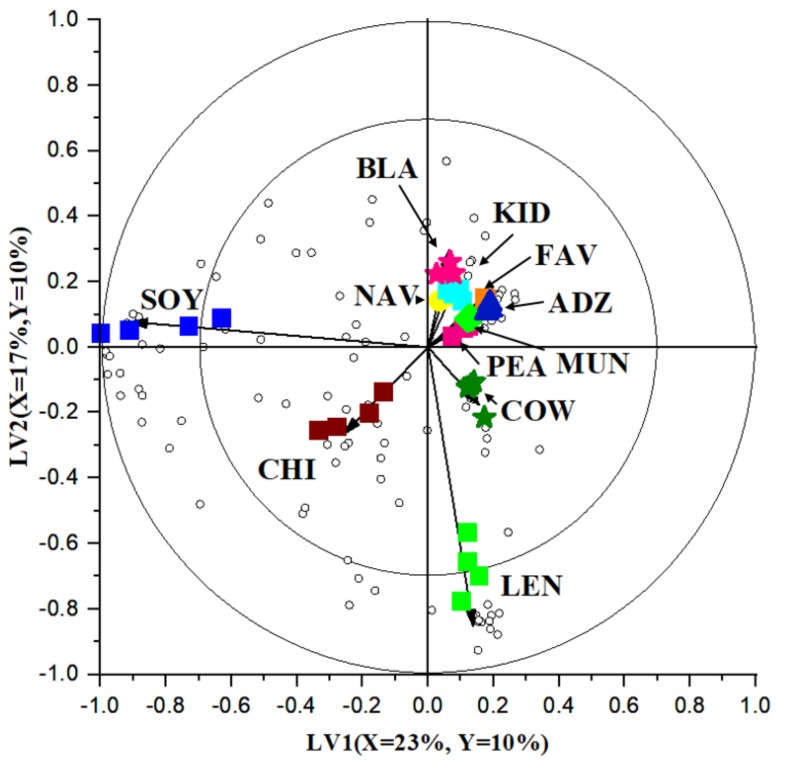
A bi-plot based on partial least square discriminant analysis (PLS-DA) comparing the volatile and fatty acid profiles among the 11 types of legumes. The variance explained is (X = 22%, Y = 10%) and (X = 17%, Y = 10%) for the first and second latent variable, respectively. 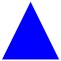 = Adzuki bean (ADZ) A. 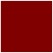 = Chickpea (CHI) 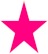 = Black bean (BLA) 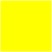 = Navy bean (NAV) 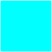 = Kidney bean (KID) 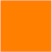 = Fava bean (FAV) 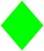 = Mung bean (MUN) 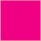 = Pea (PEA) 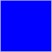 = Soybean (SOY) 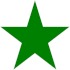 = Cowpea (COW) 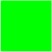 = Orange lentil (LEN).

Thereafter, a partial least squares discriminant analysis (PLS-DA) model was constructed using the volatile and fatty acid profiles as X-variables and the 11 types of legumes as categorical Y-variables. A bi-plot constructed using the first two latent variables (LVs) is shown in Figure 2. On the bi-plot, samples that are close to each other are considered similar, whereas samples that are further apart are considered different [18]. Figure 2 clearly shows that soybean and chickpea are projected further away from other samples in their own quadrant, indicating a large difference compared to the other legumes. The third quadrant is shared by cowpea and lentil, again indicating differences from other legumes. The fourth quadrant is occupied by pea, mung bean, fava bean, adzuki bean and all three *Phaseolus* beans, indicating similarity between the samples, especially between kidney, navy and black beans. This similarity may be attributed to them belonging to the same species. 

**Figure 3:**
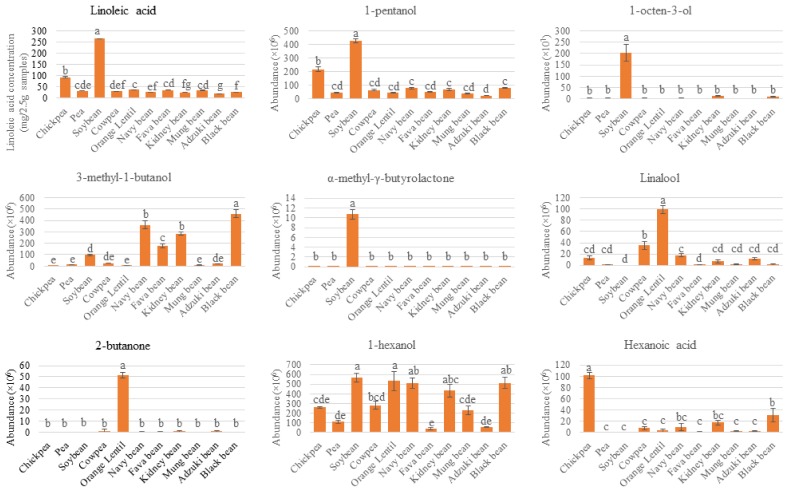
Individual plots of some representative discriminant compounds driving the distinction between legume samples observed in the PLS-DA biplot in Figure 2. Result expressed as mean ± standard deviation of four independent measurements per legume type (*n* = 4). Samples with different letters indicate significant difference (*p* < 0.05).

In addition to the legume samples, unfilled circles on the bi-plot represent volatile and fatty acid compounds (X-variables). The location of each circle represents its relation to other measured attributes (X-variables) or samples (Y-variables). Hence, a PLS-DA bi-plot provides a graphical representation of the relation between measured attributes and legume types. To gain further understanding into the specific volatiles or fatty acids which are clearly different between legume samples, variable selection was performed using a VID technique. The selected discriminant compounds are listed in Table 2. To illustrate the differences amongst the seeds, some representative discriminant volatiles and fatty acids are also visually presented in Figure 3, with significant difference (*p* < 0.05) determined using analysis of variance and Tukey’s post-hoc test. Key points are discussed in Section 4.

## 4. Discussion

The information gathered in this study has shown some key trends emerging from data analysis. These subsections discuss them in relation to the existing literature and known reaction pathways.

### 4.1. Ratio of Unsaturated Fatty Acids in Studied Legumes

The *Phaseolus* group (navy, kidney and black bean) has a low ratio (<1) of omega-6 (n-6; linoleic acid) to omega-3 (n-3; α-linoleic acid) fatty acids. Adzuki bean, mung bean and cowpea also have relatively low n-6/n-3 ratio. This is in contrast to chickpea and fava bean, which have a very high n-6/n-3 ratio of more than 18 and 14, respectively (Table 1). Diets where the ratio of fat consumed has a high n-6/n-3 ratio (≥10:1) have been linked to risk of developing chronic noncommunicable diseases, such as autoimmune and inflammatory disease, cardiovascular disease and cancer, whereas a ratio of less than 4:1 generally indicates better health outcomes [21]. Therefore, legumes, excluding chickpea and fava bean, are a good source of essential fatty acid with a desirable n-6/n-3 ratio.

However, despite their health benefits, unsaturated fatty acids are known to be more susceptible to oxidation than saturated fatty acids, due to the presence of one or more double bonds. The extent of lipid oxidation in foods can be reliably measured by determining the peroxide value so that preventive measures can be taken to delay oxidation in legume seeds after harvest while preventing the production of undesirable rancid flavour in legumes [22]. Endogenous enzymes present in legumes such as lipoxygenase utilise unsaturated fatty acids as substrate, producing volatile compounds, some of which possess undesirable odours. This is especially a problem for legumes with a high proportion of unsaturated fatty acids (>80%), such as soybean and chickpea (Table 1). The evolution and presence of volatile compounds are discussed further in the next section.

### 4.2. Aldehydes, Alcohols, Ketones and Terpenes in Studied Legumes

Aldehydes were the most abundant chemical class detected in all samples, except for navy, kidney and black beans; these beans seem to be rich in alcohols. Hexanal was the most abundant compound detected in all samples, except for navy bean. Nonanal and 2-hexenal were the second and third most abundant aldehyde in the legumes studied in this work. This is consistent with previous studies in soybean, winged bean [23] and three cultivars of common beans (black, pinto and dark red kidney) [10]. The difference in hexanal abundance may be attributed to the difference in the amount of linoleic and linolenic acid (Table 1), since they are the precursor for lipoxygenase-catalysed evolution of hexanal [3].

Lipoxygenases have been well characterised for soybean and chickpea [24]. Soybean and chickpea lipoxygenase isozymes form C_9_ and C_13_ hydroperoxides of PUFAs, acting on linoleic and α-linolenic acid, while chickpea lipoxygenase also exhibited high co-oxidation with carotene and retinol compounds [25]. Hydroperoxide lyase isozymes then cleave the aforementioned C9 and C13 hydroperoxides into isomeric nonenals, 4-hydroxynonenal and 9-oxo-nonanoic acid [26] and hexanal, cis-3-hexenal and 12-oxo-cis-9-dodecenoic acid [27,28], respectively. These products can degrade and/or be further acted on by isomerase and other enzymes, generating additional volatile aldehydes [29], which may help to explain the presence of other analogous aldehydes such as pentanal, heptanal, octanal, 2-hexenal, 2-heptenal and 2-octenal in soybean and chickpea.

Alcohols comprised the second-most abundant chemical class of volatile compounds in the headspace of legume seeds, except for navy bean, where hexanol is the most abundant volatile compound. This is not surprising, as legumes contain alcohol dehydrogenases that act on products of the lipoxygenase pathway described above. For example, three isozymes of alcohol dehydrogenase have been described in chickpea, which catalyse the interconversion of aldehydes, alcohol and acid [30], possibly explaining the abundance of 1-hexanol and 1-pentanol in chickpea. Other alcohols prominent in legumes possibly arising from enzymatic actions include 1-penten-3-ol and 1-octen-3-ol, two compounds which had been described as having undesirable odour.

Ketones and hydrocarbons are also detected in the headspace volatile fractions of legumes, though less so than alcohol and aldehydes. Ketones and hydrocarbons are also derived from lipid oxidation, from both (non) enzymatic oxidative degradations [2]. For example, 2,3-pentanedione is found in oxidised soybean oil and has a buttery flavour (Seals and Hammond 1970). Acetone, 6-methylhept-5-en-2-one (methyl heptenone) and (E,E)-3,5-octadien-2-one detected in this study have been found in large quantities in dry beans [10].

Terpenes were also detected in the headspace volatile of legume seeds; the largest number is detected in lentils. Unlike aldehydes, alcohols, ketones and hydrocarbons, which are products of fatty acid oxidation, terpenes are naturally present/synthesised by the plant [2]. α-Pinene and β-pinene are the two most common terpenes in legume samples investigated.

### 4.3. Lipoxygenase is the Most Substantial Contributor for Volatile Evolution

Out of the 11 samples, soybean contains the highest number (18) of discriminating compounds (including volatiles and fatty acids), and this can also be seen on the bi-plot (Figure 2) as soybean samples are projected the furthest away from other legumes. Volatiles with high VID coefficients in soybean comprise aldehydes (e.g., heptanal), ketones (e.g., 1-octen-3-one) and alcohols (e.g., 1-octen-3-ol and 1-pentanol). In literature, these compounds are associated with undesirable odours in soybean. For example, 1-pentanol has a pungent fusel or solvent-like odour, while 1-octen-3-ol has a mushroom and earthy odour with a low detection threshold at 1 ppb in water and 1 ppm in soymilk [31]. Individual plots presented in Figure 3 illustrate their intensity/abundance in comparison to other legume samples. These compounds are products of lipoxygenase-catalysed fatty acid oxidation. Linoleic acid is a precursor of many aldehydes, ketones and alcohols, as it is susceptible to autoxidation and lipoxygenase-catalysed oxidation [3]. Accordingly, linoleic, stearic, palmitic and oleic acids are also detected in a higher amount in soybean, in descending order (Table 2 and Figure 3). This shows the potential of the multiplatform approach followed by chemometrics, such as multivariate data analysis, to demonstrate relationships amongst different measured attributes—in this case, fatty acids and volatile profile.

However, other legume seeds also contain fatty acids and their own isozymes of lipoxygenases [24]. Even a low amount of fatty acid may generate volatiles contributing to off-odours [4,32,33]. Therefore, while it holds that lipoxygenase-catalysed fatty acid oxidation products are detected in much higher abundance in soybean because of its high linoleic acid (and other fatty acid) content, these volatiles can also be present in other legume seeds.

### 4.4. Soybean and Cowpea Contain Distinctive Butyrolactones

Three butyrolactone compounds were selected as discriminant compounds in soybean (α-methyl-γ-butyrolactone and β-methyl-γ-butyro-lactone) and cowpea (γ-ethyl-γ-butyrolactone). β-scission of fatty acid hydroperoxides can yield carbonyl compounds, which can participate in Maillard and Strecker degradation reactions to yield the aforementioned cyclic volatile compound [34]. Perhaps it is due to the combination of lipoxygenase isozyme specific to soybean, combined with plentiful precursor (Table 1), that α-methyl-γ-butyrolactone was able to be detected exclusively in soybean (Figure 3). As for the other three lactones, their presence in *Phaseolus* (β-methyl-γ-butyro-lactone) and all samples except for fava bean (γ-ethyl-γ-butyrolactone) indicates the presence of lipoxygenase with similar specificity. This is supported by Chigwedere, Tadele, Yi, Wibowo, Kebede, Van Loey, Grauwet and Hendrickx [15], with β-methyl-γ-butyro-lactone also detected in *Phaseolus* samples, whereby it was identified as a marker distinguishing aged and fresh beans cooked for 270 min.

### 4.5. Orange Lentil Contains Discriminant Terpene and Carotenoid Degradation Products

Based on Figure 2, orange lentil has a distinct volatile and fatty acid profile compared to other legumes, with 15 discriminant volatile compounds. Specifically, orange lentil is distinct for the presence of a high number of discriminating terpenes, terpene derivatives and cyclic hydrocarbons. This result is not surprising, as the orange colour of the lentil suggests the presence of carotenoids. A previous study conducted by Zhang, et al., [35] showed that the total carotenoid content of 20 cultivars of red lentil grown in Canada by dry weight ranged between 5.32 and 28.1 μg/g. In addition to being secondary metabolites, volatile terpenes may arise from degradation of carotenes by either legume lipoxygenases or hydroperoxides generated from autolytic and enzyme-catalysed lipid oxidation [2]. Linalool (Figure 3) and D-limonene can also be considered as potential discriminant compounds for orange lentil, though they were also present in other samples. These volatiles are associated with a citrus and fresh odour [36].

### 4.6. Presence of 2-Butanone and Methylated Compounds in Orange Lentil

In orange lentils, 2-butanone and pyrrole were selected as discriminant compounds with high VID coefficients (Table 2). The ketone 2-butanone has a “moderately sharp, sweet, pungent, and acetone-like” odour and has been reported as a product of lipoxygenase-catalysed oxidation of unsaturated fatty acid hydroperoxides [37], as well as temperature-accelerated oxidation of saturated fatty acids [3]. According to individual plot (Figure 3), a high relative amount of 2-butanone was detected in orange lentil in the current study and has previously been reported in lentils, navy bean, red kidney bean and peas [2,12,37]. With orange lentil only having 3.9% lipid content (Table 1), the high relative abundance of 2-butanone suggests that the sample may have undergone heat treatment in its distribution chain, or that some components of orange lentil are especially vulnerable to heat treatment. This hypothesis is supported by the presence of pyrrole. Ma, Boye, Azarnia and Simpson [37] similarly reported pyrrole in navy bean, red kidney bean, green lentil and yellow pea that had undergone heat treatment.

### 4.7. Presence of Acid in Chickpea and Fava Bean Suggests Alcohol Dehydrogenase Activity

Hexanoic acid is selected as a discriminant compound in chickpea (Table 2). This compound has been linked with the alcohol dehydrogenase pathway, which converts alcohol (in this case, 1-hexanol) into the corresponding acid [38]. From a visual inspection of the individual plots of 1-hexanol and hexanoic acid (Figure 3), chickpea only had a moderate amount of 1-hexanol, but it had the highest amount of hexanoic acid detected. This seems to suggest that alcohol dehydrogenase was present in a high concentration or had relatively high activity compared to other isozymes. A similar observation can be seen with fava bean, where it had a distinctly higher relative amount of 3-methyl-butanoic acid (VID coefficient 0.863), corresponding to 3-methyl-1-butanol contributing to 19.1% of its headspace volatile. Similar to lipoxygenase, alcohol dehydrogenase also has isozymes. Gomes, Jadrić, Winterhalter and Brkić [30] have reported three isozymes in chickpea cotyledon. This could suggest that chickpea and fava bean contain alcohol dehydrogenase which converts aldehydes to alcohols.

### 4.8. Members of the Phaseolus Group Appears to Contain Similar Dominant Volatile Compounds

In this study, 3-methyl-1-butanol was identified as a discriminant compound common to kidney, black and navy beans. Figure 3 visually illustrates its abundance. As previously stated in Section 3.3, hexanal and 1-penten-3-ol are volatile common to the *Phaseolus* legumes and have previously been identified as marker compounds in three cultivars of common beans, having high scores in the principle components [10]. This indicates that despite being of different cultivars and colours, kidney, navy and black beans have similar characterising headspace volatiles.

## 5. Conclusions

The approach used in this paper, integrating fingerprinting and profiling, is effective to characterise the volatile and fatty acid profiles of the eleven legumes seeds selected. The detected volatiles can be grouped into aldehyde, alcohol, ketone, terpene, ester and lactone and hydrocarbon chemical classes. The lipid profiles comprised palmitic, stearic, oleic, linoleic and α-linolenic acids. Advanced chemometrics utilising multivariate data analysis were used to determine distinctive volatile compounds for different legume species. The occurrence of specific discriminant compounds is hypothesised to be majorly derived from the action of species-specific isozymes, especially lipoxygenases. While findings from this result majorly emphasised how certain volatile compounds discriminate each legume and how they are linked to fatty acids, this information may aid in choosing legume-based ingredients with the desired volatile profile. This insight can also be valuable to legume breeders in selecting legumes with certain fatty acid profile, aiming for higher n-6/n-3 ratios and/or oxidative stability. Lastly, since legumes are typically processed (such as soaking and cooking) prior to consumption, the effects of processing on these legume-specific volatiles is worth further investigation.

## Figures and Tables

**Table 1:** Relative fatty acid abundance of 11 types of legume seeds, as analysed by fatty acid methyl ester gas chromatography coupled with a flame ionisation detector (FAME-GC-FID).

Legumes	Total Lipid Extracted (g/100g Sample)	C16:0 (g/100g Lipid)	C18:0 (g/100g Lipid)	C18:1 (g/100g Lipid)	C18:2n-6 (g/100g Lipid)	C18:3n-3 (g/100g Lipid)	SFAs (g/100g Lipid)	MUFAs (g/100g Lipid)	PUFAs (g/100g Lipid)	n-6/n-3 Ratio
Soybean	19.20 ^d^ ± 1.98	11.76 ^a^ ± 0.13	3.68 ^c,d^ ± 0.01	19.20 ^c^ ± 0.28	55.15 ^g^ ± 0.15	8.88 ^b^ ± 0.02	15.44 ^a,b^ ± 0.13	19.20 ^c^ ± 0.28	64.03 ^c,d^ ± 0.14	6.21 ^c^ ± 0.02
Chickpea	7.73 ^c^ ± 0.73	10.94 ^a^ ± 0.20	1.80 ^a^ ± 0.0.16	37.87 ^e^ ± 0.16	45.78 ^f^ ± 0.42	2.33 ^a^ ± 0.05	12.74 ^a^ ± 0.35	37.87 ^e^ ± 0.16	48.11 ^a^ ± 0.46	19.67 ^e^ ± 0.36
Lentil	3.90 ^a,b^ ± 0.21	21.40 ^e^ ± 0.41	2.77 ^a,b,c^ ± 0.10	28.06 ^d^ ± 0.35	38.21 ^c,d^ ± 0.55	9.07 ^b^ ± 0.09	24.29 ^e^ ± 0.40	28.06 ^d^ ± 0.35	47.27 ^a^ ± 0.64	4.21 ^b,c^ ± 0.03
Cowpea	3.46 ^a,b^ ± 0.10	27.68 ^f^ ± 0.26	4.76 ^e^ ± 0.53	7.35 ^a,b^ ± 1.64	35.97 ^b,c^ ± 0.85	23.34 ^e^ ± 0.48	33.34 ^f^ ± 0.97	7.35 ^a,b^ ± 1.64	59.31 ^b,c^ ± 1.05	1.54 ^a^ ± 0.04
Pea	3.41 ^a,b^ ± 0.12	13.48 ^a,b^ ± 0.35	4.50d ^e^ ± 0.11	34.40 ^e^ ± 2.30	38.66 ^c,d^ ± 2.38	8.78 ^b^ ± 0.67	17.98 ^b,c^ ± 0.44	34.40 ^e^ ± 2.30	47.44 ^a^ ± 3.04	4.41 ^b,c^ ± 0.07
Mung bean	3.20 ^a,b^ ± 0.15	27.04 ^f^ ± 1.56	5.73 ^f^ ± 0.12	6.54 ^a^ ± 37.78	43.71 ^e,f^ ± 2.23	15.82 ^c^ ± 1.17	33.75 ^f^ ± 2.22	6.54 ^a^ ± 3.78	59.53 ^b,c,d^ ± 3.18	2.76 ^a,b^ ± 0.14
Fava bean	2.75 ^a,b^ ± 0.19	15.25b ^c^ ± 0.39	3.64 ^c,d^ ± 0.93	24.57 ^c,d^ ± 0.34	52.68 ^g^ ± 0.88	3.61 ^a^ ± 0.57	19.14 ^c,d^ ± 1.02	24.57 ^c,d^ ± 0.34	56.29 ^b^ ± 1.17	14.59 ^d^ ± 2.06
Navy bean	3.87 ^b^ ± 0.17	18.00 ^d^ ± 0.22	3.12 ^bc^ ± 0.13	19.49 ^c^ ± 0.81	28.30 ^a^ ± 0.45	31.09 ^f^ ± 0.33	21.12 ^d^ ± 0.15	19.49 ^c^ ± 0.81	59.39 ^b,c,d^ ± 0.73	0.91 ^a^ ± 0.01
Kidney bean	3.59 ^a,b^ ± 0.14	17.97 ^d^ ± 0.49	2.40 ^a,b^ ± 0.27	12.71 ^b^ ± 2.00	29.02 ^a^ ± 1.16	35.71 ^g^ ± 1.39	20.49 ^c,d^ ± 0.65	12.71 ^b^ ± 2.00	64.74 ^d^ ± 2.55	0.81 ^a^ ± 0.00
Black bean	3.20 ^a,b^ ± 0.16	17.17c ^d^ ± 0.48	2.39 ^a,b^ ± 0.29	8.29 ^a,b^ ± 0.16	33.65 ^b^ ± 0.34	37.30 ^g^ ± 1.22	19.56 ^c,d^ ± 0.71	8.29 ^a,b^ ± 0.16	70.95 ^e^ ± 1.50	0.90 ^a^ ± 0.02
Adzuki bean	1.96 ^a^ ± 0.10	27.96 ^f^ ± 1.02	3.36 ^c^ ± 0.40	3.92 ^a^ ± 0.52	41.88 ^d,e^ ± 1.12	20.31 ^d^ ± 0.73	31.35 ^f^ ± 1.45	3.92 ^a^ ± 0.52	62.19 ^c,d^ ± 1.96	2.06 ^a^ ± 0.01
F-value	170.887	229.2	33.7	93.3	114.7	1047.5	148.5	93.3	46.0	218.0
Significant	0.00	0.00	0.00	0.00	0.00	0.00	0.00	0.00	0.00	0.00

Values expressed as mean ± standard deviation (*n* = 4). C16:0 = palmitic acid; C18:0 = stearic acid; C18:1 = oleic acid; C18:2 = linoleic acid; C18:3 = α-linolenic acid; SFAs = saturated fatty acids; MUFAs = monounsaturated fatty acids; PUFAs = polyunsaturated fatty acid; n-6/n-3 = ratio of omega-6 to omega-3 fatty acids. Means with different superscripts in the same column indicate significant difference (*p* < 0.05).

**Table 2:** List of discriminant volatile compounds/fatty acids for individual legume samples.

VID	Identity	RI	Chemical Group
Soybean (18)			
0.989	α-methyl-γ-butyrolactone	1621	Ester & Lactone
0.977	1-octen-3-one	1321	Ketone
0.971	β-methyl-γ-butyro-lactone	1644	Ester & Lactone
0.967	Heptanal	1189	Aldehyde
0.964	Linoleic acid	*	Fatty Acid
0.959	2(*Z*)-heptenal	1349	Aldehyde
0.958	Stearic acid	*	Fatty Acid
0.941	1-octen-3-ol	1461	Alcohol
0.934	1-pentanol	1251	Alcohol
0.934	2(*E*)-octenal	1458	Aldehyde
0.920	Palmitic acid	*	Fatty Acid
0.902	3,5-octadien-2-ol	1433	Alcohol
0.889	2(*Z*)-penten-1-ol	1330	Alcohol
0.878	2,4-nonadienal	1710	Aldehyde
0.874	5-ethylcyclopent-1-enecarboxaldehyde	1451	Aldehyde
0.867	3-octanone	1268	Ketone
0.823	Pentanal	949	Aldehyde
0.804	Oleic acid	*	Fatty Acid
Lentil (15)			
0.984	2-butanone	873	Ketone
0.982	Pyrrole	1540	Pyrrole
0.976	Menthol	1647	Alcohol
0.974	2-methoxyethylbenzene	1519	Hydrocarbon
0.970	Anethole	1816	Hydrocarbon
0.966	Caryophyllene	1630	Terpene
0.958	o-cymene	1291	Terpene
0.953	α-copaene	1529	Terpene
0.944	Linalool	1554	Terpene
0.941	Terpinen-4-ol	1620	Terpene
0.898	α-terpinyl acetate	1705	Terpene
0.892	γ-Terpinene	1262	Terpene
0.820	p-Cymen-7-ol	2009	Terpene
0.819	D-limonene	1208	Terpene
0.806	2(*E*)-hexenal	1230	Aldehyde
Chickpea (8)			
0.967	Allyl nonanoate	1632	Ester & Lactone
0.943	1,4-dichlorobenzene	1478	Hydrocarbon
0.884	6-methyl-5-hepten-2-one	1358	Ketone
0.883	1-octanol	1564	Alcohol
0.883	Nonanal	1418	Alcohol
0.859	Hexanoic acid	1831	Acid
0.833	2(*E*)-decenal	1657	Aldehyde
0.800	6-methyl-3,5-heptadiene-2-one	1613	Ketone
Cowpea (6)			
0.975	α-muurolene	1734	Hydrocarbon
0.971	4,1 methylethyl benzaldehyde	1785	Aldehyde
0.967	α-terpinen-7-al	1796	Aldehyde
0.953	γ-ethyl-γ-butyrolactone	1719	Ester & Lactone
0.925	3-p-menthen-7-al	1598	Aldehyde
0.803	γ-methyl-γ-butyrolactone	1639	Ester & Lactone
Mung bean (5)			
0.967	p-xylene	1194	Hydrocarbon
0.954	Isophorone	1624	Ketone
0.900	o-xylene	1133	Hydrocarbon
0.877	1,3-dimethylbenzene	1141	Hydrocarbon
0.868	Toluene	1019	Hydrocarbon
Fava bean (2)			
0.904	7-epi-silphiperfol-5-ene	1490	Ester & Lactone
0.863	3-methylbutanoic acid	1684	Acid
Pea (1)			
0.875	2-pentanone	947	Ketone
Adzuki bean (6)			
0.934	3-furaldehyde	1455	Aldehyde
0.925	3-furanmethanol	1680	Furan
0.846	2-methylfuran	869	Furan
0.810	Methyl salicylate	1782	Ester & Lactone
Kidney bean (1)			
0.838	3-methyl-1-butanol	1202	Alcohol
Black bean (5)			
0.900	β-pinene	1099	Terpene
0.873	α-pinene	997	Terpene
0.869	3-methyl-1-butanol	1202	Alcohol
0.859	4(*Z*)-heptenal	1254	Aldehyde
0.847	1-penten-3-ol	1148	Alcohol
Navy bean (1)			
0.871	3-methyl-1-butanol	1202	Alcohol

* Experimental retention index was calculated for each volatile compound, while individual fatty acids were identified by matching retention time with commercial standards (FAMQ-005, AccuStandards, New Haven, CT, USA).

## Supplementary Materials

The following are available online at https://www.mdpi.com/2304-8158/8/12/651/s1, Table S1: Pure standards injected to confirm identity of selected compounds of interest (10 ppm).
