# Supplementary material for: A Comprehensive Characterisation of Volatile and Fatty Acid Profiles of Legume Seeds
Source: Foods. 2019 Dec 6;8(12):651. doi: 10.3390/foods8120651 (PMC6963610; doi:10.3390/foods8120651)
Supplement: Supplementary file 1 [file foods-08-00651-s001.pdf]

Supplementary Materials

Table 1. Pure standards injected to confirm identity of selected compounds of interest (10ppm).

Specific compounds	Chemical Class	Alternative Name
Hexanoic acid	Acid	
1-hexanol	Alcohol	
1-octen-3-ol	Alcohol	
3-hexen-1-ol	Alcohol	
3-methyl-butanol	Alcohol	
2(Z)-heptenal	Aldehyde	
2(E)-octenal	Aldehyde	
2(E)-decanal	Aldehyde	
Hexanal	Aldehyde	
2(E)-hexenal	Aldehyde	
Benzaldehyde	Aldehyde	
3-methyl-butanal	Aldehyde	
2-methyl-butanal	Aldehyde	
2(E), 4(E)-heptadienal	Aldehyde	
3-furaldehyde	Aldehyde	
Anethole	Hydrocarbon	p-propenyl-anisole
dihydro-3-methyl-2(3h)-furanone	Esters & Lactones	α-Methyl-γ-butyrolactone
dihydro-4-methyl-2(3h)-furanone	Esters & Lactones	β-Methyl-γ-butyro-lactone
β-Pinene	Terpene	
D-Limonene	Terpene	
Linalool	Terpene	
o-Cymene	Terpene	
γ-Terpinene	Terpene	
